# Nitrendipine-Treatment Increases Cork Spot Disorder Incidence in Pear ‘Akituki’ (*Pyrus pyrifolia* Nakai.) by Altering Calcium Distribution Inside the Fruit

**DOI:** 10.3390/plants10050994

**Published:** 2021-05-17

**Authors:** Zhenhua Cui, Nannan Wang, Dingli Li, Ran Wang, Chunhui Ma

**Affiliations:** College of Horticulture, Qingdao Agricultural University, Qingdao 266109, China; zhcui@qau.edu.cn (Z.C.); wangnannan2001@163.com (N.W.); lidingli@qau.edu.cn (D.L.); rwang@qau.edu.cn (R.W.)

**Keywords:** *Pyrus*, cork spot disorder, calcium deficiency, nitrendipine

## Abstract

‘Akituki’ (*Pyrus pyrifolia* Nakai.) is a very popular and profitable pear cultivar in China. However, its high susceptibility to cork spot disorder has limited its expansion of cultivated area. The mechanisms of cork spot disorder have been discussed extensively, focusing on Ca^2+^ deficiency, yet no consensus has been made. In this study, we applied nitrendipine (NI) as a Ca^2+^ uptake inhibitor to explore the role of calcium in cork spot disorder occurrence. Results showed that NI treatment on the fruit remarkably increased the incidence of cork spot disorder; alteration of mineral contents happened at the early developmental stage of the fruit, especially on the outer flesh and the peel of the fruit; and this gap was filled gradually along with the expansion of the fruit. Significant differences in the expression levels of Ca^2+^ transport-related genes were found in the inner flesh, outer flesh and peel during the fruit growth period. The observation of free Ca^2+^ localization indicated the intracellular imbalance of Ca^2+^ in the NI-treated fruit. In conclusion, NI treatment reduced the calcium content in the fruit at an early developmental stage, altered the related expression of genes and influenced the cellular Ca^2+^ balance in the fruit, which prompted the occurrence of cork spot disorder. Measures for the prevention and control of cork spot disorder should be taken at the early stage of the fruit development in the field.

## 1. Introduction

Cork spot is a physiological disorder of fruit which occurs most in pears and apples. The typical characteristics of this disorder in pears are brown desiccated flesh or grayish corky lesions beneath the fruit skin [1], and a bumpy fruit surface in some cultivars [2]. This disorder in pears initiates in the early developmental stage of the fruit and continues to harvest time, depending on cultivar specificity [3]. ‘Akituki’ (*Pyrus pyrifolia*), bred in Japan in 1988, is a very popular cultivar in China for its large fruit size, pretty fruit shape and taste. However, ‘Akituki’ is very susceptible to cork spot disorder [1], which has caused great economic loss in pear industry and has become a major obstacle to the continuing expansion of the cultivated area [1]. However, the mechanisms of cork spot disorder remain poorly understood; therefore, the management techniques to prevent this disorder are often inadequate.

Similar to bitter pits [4] of apples, and hard ends [5] and superficial scalding [6] of pears, cork spot disorder was generally believed to be closely related to the content of Ca [7,8,9]. Foliar spraying of exogenous Ca^2+^ solution three to five times during the growing period could effectively reduce the incidence of cork spot disorder in ‘Anjiu’ pears [10,11,12]. Therefore, cork spot was believed to be a Ca^2+^-deficiency-related physiological disorder. However, a recent study found that spraying a calcium solution, a boron solution or a mixture solution of calcium and boron during the growing period could not reduce the incidence of cork spot-like physiological disorder in apples, and there was no correlation between the incidence of cork spot-like physiological disorder and Ca/B content in fruit or leaves [13]. Interestingly, higher levels of total calcium were found in cork spotted ‘Akituki’ [1] and ‘Chili’ [2] pear fruit than healthy fruit. These findings suggested that calcium plays an important role in cork spot disorder, but its mechanism is unclear and needs further exploration.

When investigating the effect of Ca^2+^ on cork spot disorder, a supplement of exogenous calcium was applied in most experimental designs [14], but a deliberate calcium deficit was rarely induced to analyze the effect of calcium in cork spot development. Nitrendipine (NI), as an inhibitor of the Ca^2+^ transport channel, is mainly used to treat heart disease [15]. In plants, NI can inhibit the movement of Ca^2+^ through the L-type Ca^2+^ channel in lilies [14] and block the entry of outer Ca^2+^ into carrot callus cells [16]. It also has been shown to completely inhibit the entry of Ca^2+^ into peanut pod cells, and this inhibition was reversible when NI treatment was removed [14,17]. Therefor NI is a suitable reagent for studying the impact of Ca^2+^ deficit on cork spot development by inhibiting the absorption of Ca^2+^ in pear fruit.

The objective of the present study was to investigate the impact of Ca^2+^ deficit on the cork spot disorder occurrence. NI was applied in the study to inhibit the Ca^2+^ absorption in pear fruit, followed by the measurement of mineral content and the expression levels of Ca^2+^ transport-related genes, and the localization of free Ca^2+^. The relationship between Ca^2+^ deficiency and cork spot incidence was also analyzed. Results of the study are expected to provide valuable clues for the exploration of the cork spot disorder’s mechanism and to be useful for the control of cork spot disorder in orchards.

## 2. Results

### 2.1. The Effects of NI Treatment on Cork Spot Incidence and Fruit Quality

**Figure 1:**
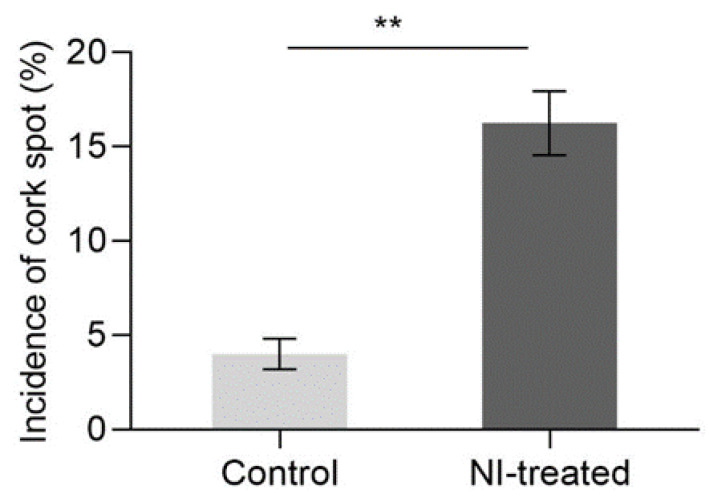
The comparison of cork spot incidence between NI-treated fruit and control fruit. Data were collected from 2019 to 2020 and are presented as means ± SE; ** means significant difference at *p* < 0.01 level.

**Figure 2:**
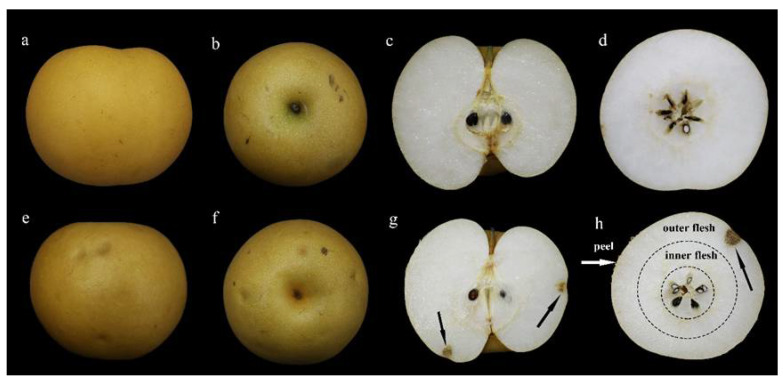
Observation of the cork spot symptom at harvest time (127 DAFB). (**a**–**d**) are healthy fruit; (**e**–**h**) are cork spotted fruit; black arrows indicate the cork spotted tissues.

For a two-year consecutive investigation, the cork spot incidence of NI-treated fruit at harvest time was much higher (16%) than that of the control fruit (4%) (Figure 1). The cork spotted fruit had slight pitting on the surface compared with the healthy fruit (Figure 2a,b,e,f). For the anatomical observation, the cork spots in disordered fruit distributed close to the distal end of the fruit longitudinally and on the outer flesh position of the equatorial cutting surface, respectively (Figure 2g,h). The inner flesh and the fruit core area rarely showed cork spot distribution (Figure 2g,h). For the fruit treated with NI not showing cork spot symptoms, the fruit size had no change compared with the control fruit, including the single fruit weight, vertical length and horizontal length (Table 1). Similar levels of total soluble solid, titratable acidity and fruit firmness were also found between the NI-treated fruit and the control fruit (Table 1).

### 2.2. The Effect of NI Treatment on Mineral Elements Content

**Figure 3:**
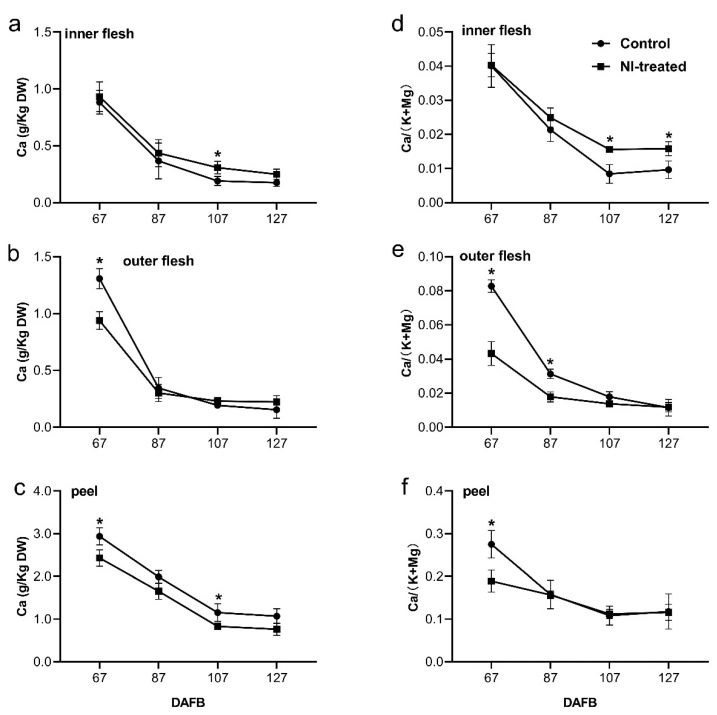
Analysis of the mineral elements of NI-treated fruit and control fruit during the fruit growth period. (**a**–**c**) are Ca content of different positions as indicated in Figure 2; (**d**–**f**) are ratios of Ca to (K + Mg) content of different positions as indicated in Figure 2. Data are presented as means ± SE; * means significant difference between control fruit and NI-treated fruit at the corresponding timepoint at *p* < 0.05 level.

According to the cork spot distribution pattern (Figure 2), the fruit inner flesh, outer flesh and peel were separately analyzed for their Ca, K and Mg contents during the fruit growth period. The Ca concentrations of both NI-treated and control fruit increased from 67 DAFB to 127 DAFB in all three positions measured in this study (Figure 3a–c). For the inner flesh, Ca concentration showed a similar level for NI-treated and control fruit, from 67 to 127 DAFB, with an exception, where NI-treated fruit had a higher concentration of Ca than control fruit at 107 DAFB (Figure 3a). The values of Ca/(K + Mg) were greater in NI-treated fruit than those of control fruit at both 107 and 127 DAFB (Figure 3e). For the outer flesh, NI treatment remarkably reduced the Ca concentration compared with control fruit at 67 DAFB (Figure 3b). The Ca concentration of control fruit declined to a similar level compared with NI-treated fruit, along with the fruit growth from 87 to 127 DAFB (Figure 3b). The values of Ca/(K + Mg) in control fruit at both 67 and 87 DAFB were significantly higher than those of NI-treated fruit (Figure 3e). For the fruit peel, the NI-treated and control fruit had similar dynamic patterns of Ca concentration (Figure 3c). The control fruit had higher concentrations of Ca at both 67 and 107 DAFB compared with NI-treated fruit (Figure 3c), and a significant difference in Ca/(K + Mg) was only found at 67 DAFB, with a higher value in control fruit than NI-treated fruit (Figure 3f).

### 2.3. The Effect of NI-Treatment on the Expression of Genes Related to Ca^2+^ Transport

**Figure 4:**
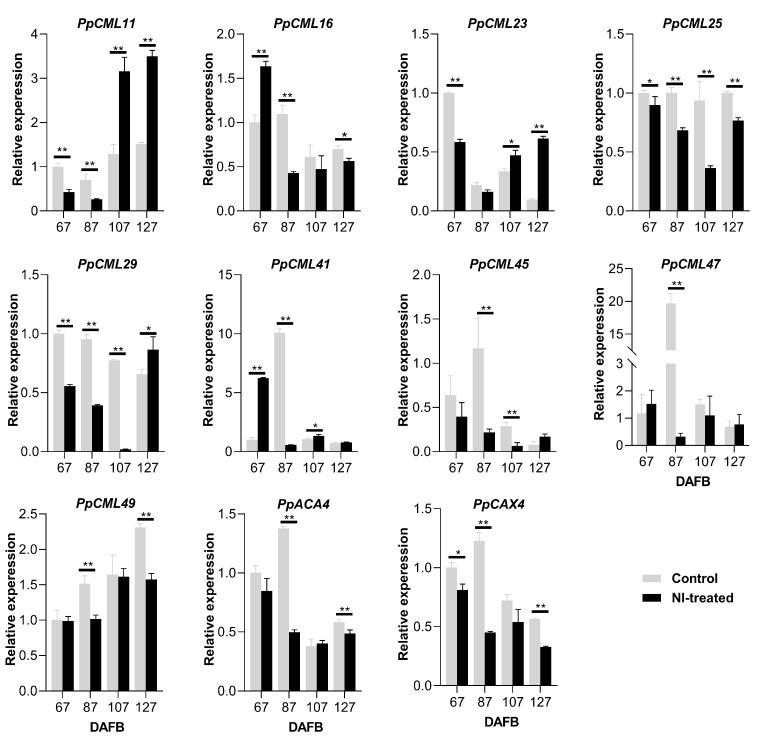
Expression analysis of Ca^2+^ transport-related genes of the inner flesh tissues during the fruit growth period. * and ** mean significantly different expression levels within the group at *p* < 0.05 and *p* < 0.01, respectively.

To further investigate the effects of NI treatment on the Ca^2+^ transport and allocation in the fruit, Ca^2+^ transport-related genes such as Ca^2+^ sensors (*PpCMLs*), Ca^2+^/H^+^ exchangers (*PpCAX*) and Ca^2^-ATPase (*PpACA*) were selected for the expression analysis. For the inner flesh, *PpCML11* had higher expression levels in control fruit than in NI-treated fruit at 67 and 87 DAFB; however, its expression increased remarkably in NI-treated fruit at 107 and 127 DAFB (Figure 4). *PpCML16* had a higher expression level in NI-treated fruit at 67 DAFB, but declined quickly from 87 to 127 DAFB. *PpCML23* had a higher expression level in control fruit at 67 DAFB, and decreased rapidly from 87 to 127 DAFB. NI-treated fruit had higher expression levels of *PpCML23* at 107 and 127 DAFB compared with control fruit. *PpCML25* expression stayed at a higher level in control fruit from 67 to 127 DAFB compared with NI-treated fruit. *PpCML29* had higher expression levels in control fruit from 67 to 107 DAFB, and its expression increased rapidly in NI-treated fruit at 127 DAFB. *PpCML41* had higher expression levels in NI-treated fruit at 67 DAFB and in control fruit at 87 DAFB, and it showed relatively lower expression levels in both NI-treated and control fruit from 107 to 127 DAFB. *PpCML45* had higher expression levels in control fruit at both 87 and 107 DAFB compared with NI-treated fruit. *PpCML47* had a drastically higher expression level at 87 DAFB in control fruit compared with NI-treated fruit. Both *PpCML49* and *PpACA4* showed higher expression levels at 87 and 127 DAFB in control fruit compared with NI-treated fruit. *PpCAX4* had higher expression levels in control fruit than that in NI-treated fruit at 67, 87 and 127 DAFB (Figure 4).

**Figure 5:**
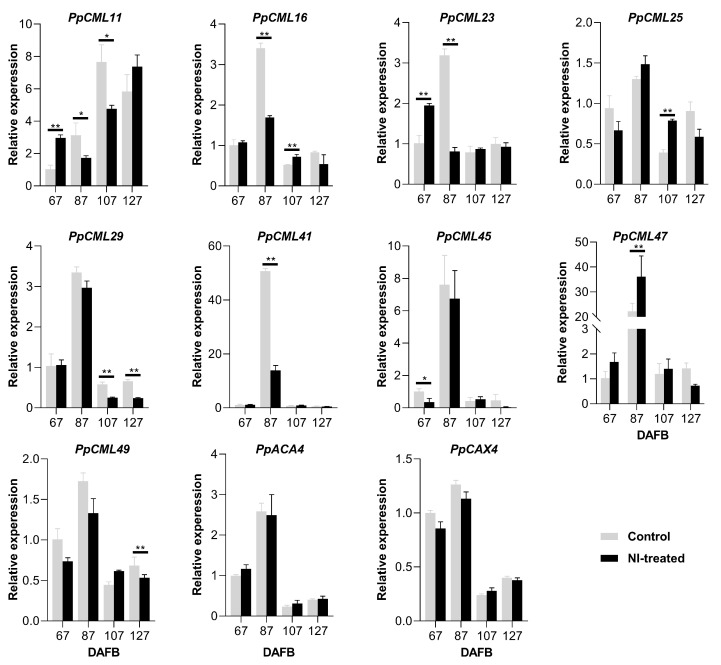
Expression analysis of Ca^2+^ transport-related genes of the outer flesh tissues during the fruit growth period. * and ** mean significantly different expression levels within the group at *p* < 0.05 and *p* < 0.01, respectively.

For the outer flesh, *PpCML11* had a higher expression level at 67 DAFB and lower expression levels at 87 and 127 DAFB in NI-treated fruit compared with control fruit (Figure 5). *PpCML16* had a remarkably higher expression level in control fruit at 87 DAFB compared with NI-treated fruit, and NI-treated fruit showed a higher expression level of *PpCML16* at 107 DAFB compared with control fruit. *PpCML23* had a higher expression level and a lower expression level at 67 and 87 DAFB, respectively, in NI-treated fruit compared with control fruit. The only significant difference in *PpCML25* expression was found at 107 DAFB with a higher level in NI-treated fruit. Both NI-treated and control fruit had high expression levels of *PpCML29* at 87 DAFB, and the control fruit had higher expression levels of *PpCML29* at both 107 and127 DAFB compared with NI-treated fruit. *PpCML41* had a far higher expression level at 87 DAFB in control fruit compared with NI-treated fruit, and very low levels of its expression were found in both control and NI-treated fruit at 67, 107 and 127 DAFB. Even though higher expression levels of *PpCML45* were only observed at 87 DAFB in both control and NI-treated fruit, a significant difference was only found at 67 DAFB with a higher expression level in control fruit. *PpCML47* showed a higher expression level in NI-treated fruit at 87 DAFB. *PpCML49* had a higher expression level in control fruit compared with NI-treated fruit at 127 DAFB. No significant difference of expression level for *PpACA4* or *PpCAX4* was found between the control fruit and the NI-treated fruit during the fruit growth period (Figure 5).

**Figure 6:**
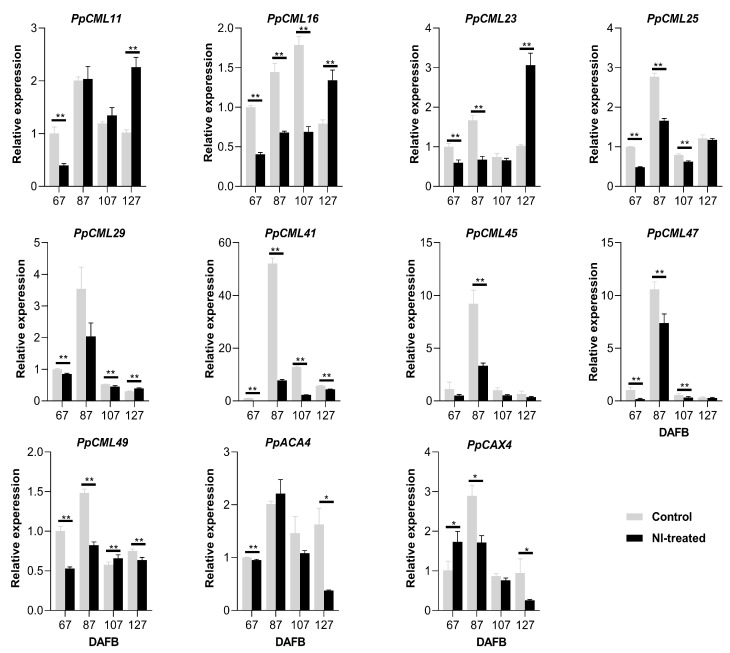
Expression analysis of Ca^2+^ transport-related genes of the fruit peel during the fruit growth period. * and ** mean significantly different expression levels within the group at *p* < 0.05 and *p* < 0.01, respectively.

For the fruit peel, *PpCML11* had a lower expression level and higher expression level in NI-treated fruit at 67 and 127 DAFB, respectively, compared with the control fruit (Figure 6). *PpCML16* showed higher expression levels in the control fruit at 67, 87 and 127 DAFB; and a higher expression level was found at 127 DAFB in NI-treated fruit. *PpCML23* had higher expression levels in control fruit at both 67 and 87 DAFB. NI-treated fruit had a higher expression level of *PpCML23* at 127 DAFB than control fruit. *PpCML25* had higher expression levels in control fruit at 67, 87 and 127 DAFB compared with NI-treated fruit. *PpCML29* had higher expression levels in control fruit at 67 and 107 DAFB, and it showed a higher expression level in NI-treated fruit at 127 DAFB. *PpCML41* kept higher expression levels in the control fruit during the whole fruit growth period compared with those of the NI-treated fruit. *PpCML45* showed a much higher expression level in control fruit at 87 DAFB compared with NI-treat fruit. *PpCML47* had higher expression levels in control fruit at 67, 87 and 127 DAFB compared with NI-treated fruit. *PpCML49* had higher expression levels in control fruit at 67, 87 and 127 DAFB; and the NI-treated fruit showed a higher expression level of *PpCML49* at 107 DAFB. *PpACA4* had higher expression levels at both 67 and 127 DAFB compared with NI-treated fruit. The NI-treated fruit showed a higher expression level of *PpCAX4* at 67 DAFB compared with control fruit. The control fruit had higher expression levels of *PpCAX4* at both 87 and 127 DAFB (Figure 6).

### 2.4. Comparison of Free Ca^2+^ Distribution

**Figure 7:**
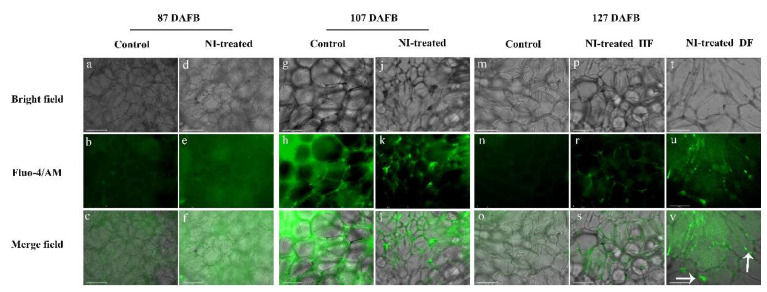
Observation of free Ca^2+^ distribution in the outer flesh tissues of control and NI-treated fruit by laser scanning confocal microscope. Green fluorescence in the merge field indicated the Ca^2+^ distribution. White arrows indicate the intensified Ca^2+^ signal; HF means healthy fruit; DF means cork spot disordered fruit; scale bars = 100 μm.

Since the cork spot distributed mostly in the outer flesh of the fruit, the free Ca^2+^ of both intercellular and intracellular were localized to analyze the Ca^2+^ distribution in the outer flesh (Figure 7). At 87 DAFB, both the control fruit and NI-treated fruit showed a homogeneous pattern of Ca^2+^ distribution, and the Ca^2+^ signal intensity was similar between them. At 107 DAFB, the Ca^2+^ signal was not as strong as that at 87 DAFB in control fruit, and the Ca^2+^ signal intensity of NI-treated fruit was even weaker compared with that of control fruit. An uneven distribution of Ca^2+^ signal was observed in NI-treated fruit at 107 DAFB, and a much stronger signal was shown at the margin of the cells and the cytoplasm had far weaker Ca^2+^ signals. At 127 DAFB, both the control healthy and NI-treated healthy fruit had very weak Ca^2+^ signals compared with the fruit at 87 and 107 DAFB. Ca^2+^ still distributed homogeneously in control fruit, and NI-treated healthy fruit showed an uneven distribution of Ca^2+^ signal with stronger intensity of Ca^2+^ at the margin of the cells. However, for the NI-treated cork spot disordered tissue, much stronger intensity of Ca^2+^ was observed in the whole cells (Figure 7).

## 3. Discussion

In the present study, we explored the relationship between calcium deficiency and cork spot disorder using NI to inhibit the absorption of calcium in the fruit of ‘Akituki,’ which is an innovative method, as far as we know, in the Ca^2+^ deficiency related disorder study. Separation of the different parts of the fruit for the analysis of mineral elements, Ca^2+^ transport-related genes expression and free Ca^2+^ distribution at different growth stages improved the reliability of the results compared with previous studies focusing on the whole fruit analysis.

In our study, even though NI treatment did not influence the fruit attributes, it apparently increased the incidence of cork spot disorder in ‘Akituki’ pears by approximately 12% (Figure 1), indicating that NI altered the Ca distribution inside the fruit, which was confirmed by the observation of free Ca^2+^ localization (Figure 7). The cork spot distributed close to the calyx-end and outer flesh of fruit (Figure 2), which was consistent with other studies of cork spot in pears [1,2] and bitter pits in apples [18]. This also suggests the abnormal distribution of calcium inside the fruit. NI-treated fruit had lower Ca content only in the outer flesh and fruit peel, and the inner flesh had similar Ca content to the control fruit (Figure 3). This may be because the inner flesh has a stronger vascular connection with the pedicel compared with the outer flesh and the peel, and Ca transport was secured in the inner flesh prior to the other parts of the fruit. As a result, the ratio of Ca/(K + Mg) had a high value in the inner fruit, but it was lower than in the outer flesh and the peel (Figure 3). Potassium is known to be involved in cell-expansion-related process [19,20], and K^+^ and Mg^2+^ are known to compete with Ca^2+^ for biding sites at the plasma membrane [21,22]. The lower Ca concentration at the later stage in our results may have been due to the high levels of K^+^ and Mg^2+^ during the rapid growth of fruit, which could have led to a reduction in fruit Ca^2+^ uptake, dilution of the Ca concentration and high susceptibility to cork spot disorder [23], and also filled the gaps in mineral element contents between control and NI-treated fruit (Figure 3). Even though the cork spot occurred only in the outer flesh and started at around 100 DAFB according to our field investigation, the disorder must initiate at an earlier stage, where the Ca concentration and the Ca/(K + Mg) values demonstrated significant differences between NI-treated and control fruit (Figure 3b,e).

The observation of free Ca^2+^ distribution showed intracellular imbalance in the NI-treated healthy fruit at 107 and 127 DAFB (Figure 7), indicating that the intracellular imbalance of Ca^2+^ happened prior to the formation of cork spots, which was accordant with the analysis of mineral element content above (Figure 3). Free Ca^2+^ was found to be enriched in the cell wall in the cork spotted pear tissues, but the total Ca content was even higher in the cork spotted tissues [1,2]. In other pear Ca^2+^ deficiency-related disorders, such as superficial scald and hard end [24,25], the disordered fruits also had higher levels of free Ca^2+^ and Ca^2+^ mainly concentrated in the cell wall; and no relationship between Ca content and cork spot-like physiological disorder was found in apples [13]. Our result showed cork spotted tissues even had a stronger Ca^2+^ signal at 127 DAFB (Figure 7), which was consistent with the results mentioned above. This could be the result of cell membrane damage and cytoplasmic leakage due to the replacing of Ca^2+^ by K^+^ and Mg^2+^ on the plasma membrane; however, the function of Ca^2+^ in stabilizing the membrane structure cannot be replaced [21,22]. Early and efficient Ca uptake was considered essential for maintaining normal Ca distribution in the distal part of the fruit and preventing the occurrence of Ca deficiency-related disorder in wax apples [26]. These findings suggested that the Ca deficiency at the early stage of fruit development could be a critical factor contributing to the cork spot development. Early Ca deficiency might influence the Ca delivery inside the fruit and the intracellular distribution in the outer flesh tissues. Therefore, the measures for the prevention and control of cork spot disorder, such as foliar spraying of a Ca^2+^ solution, should be taken as early as possible in the field.

As well as the long-distance transport of Ca^2+^ via the apoplastic path, the transmembrane transport of Ca^2+^ is essential for the intracellular balance of Ca^2+^, which was mediated by membrane transport proteins, such as CML (probable calcium-binding protein), ACA (auto-inhibited Ca^2+^-ATPase) and CAX (Ca^2+^ exchanger) [27]. In our study, NI treatment altered the expression levels of *CMLs* in different positions of the fruit during the fruit growth period (Figure 4, Figure 5 and Figure 6). In Arabidopsis thaliana, *CML* are mainly stress-responsive genes, which are up-regulated under different abiotic stresses (McCormack et al., 2015, Zhu et al., 2015). *PpCML25* and *PpCML47* were up-regulated in the outer flesh of NI-treated fruit at 107 and 87 DAFB, respectively, which were the critical timepoints for the onset of cork spot disorder. Higher expression levels of *CMLs* were also shown to associate with the occurrence of other Ca^2+^ deficiency-related disorders in pear fruit, such as superficial scald [25] and hard-end [24]. In apples, the expression levels of *ACA* and *CAX* increased as the severity of bitter pits increased [18,28]. *MdCAX11* and *MdCAX5* were also identified to bind to the tonoplast and transport Ca^2+^ from the cytoplasm to the vacuole, which increased the incidence of bitter pit [18]. Interestingly, our study found that NI-treated fruit have altered expression levels of both *PpACA4* and *PpCAX4* in the inner flesh and the peel. However, no significant alterations of *PpACA4* and *PpCAX4* expression levels were found in the outer flesh (Figure 5), which is different from other studies. The different treatment in our study may have caused such results, and the differential gene expression in the inner flesh could influence the normal expression of the genes in the outer flesh. Taken together, NI treatment altered the expression levels of Ca^2+^ transport-related genes and further promoted the incidence of cork spot in ‘Akituki’ pear.

## 4. Materials and Methods

### 4.1. Plants Material and Nitrendipine Treatment

Ten-year-old ‘Akituki’ trees grafted on the rootstock of *Pyrus calleryana*, located at 36°53′ N and 118°42′ E in Weifang, Shandong Province, China, were used for the trial. Trees, spaced at 2 m within rows and 5 m between rows, were trained with a trellis cultivation system and were managed with full nutrition and water. NI (Yuanye Biology, Shanghai, China) was dissolved in lanolin at the concentration of 0.2 mg/mL and homogeneously smeared on the fruit pedicels at 47 days after full bloom (DAFB), and solvent lanolin was used as control to smear on the fruit pedicel. Twenty fruits were selected randomly on each tree for NI treatment and control, respectively, and five trees for each trial were used for the whole trial. After NI treatment on the fruit pedicels at 47 DAFB, fruit samples were taken every 20 days until harvest for different purposes of analysis as described below.

### 4.2. Investigation of Cork Spot Incidence

Cork spot incidence of the fruits selected for the trial were investigated at harvest time (127 DAFB) from 2019 to 2020. The fruit without any cork spots on both equatorial and longitudinal planes inside the fruit were considered as healthy fruit; otherwise any cork spot observed inside the fruit was considered as cork spot disorder.

### 4.3. Analysis of Fruit Quality

The fruit quality was evaluated at harvest time by measuring the single fruit weight, vertical and horizontal length, total soluble solids, titratable acidity and fruit firmness. All the operations and calculation referred to Cui et al. [2].

### 4.4. Determination of Ca, K and Mg Contents

Fruit of 67, 87, 107 and 127 DAFB were sampled for the Ca, K and Mg content analysis. For each sample, the tissues of fruit peel, outer flesh and inner flesh were separately measured for the Ca, K and Mg contents. Briefly, fruit tissues were dried in an oven at 105 °C for 30 min, and then at 75 °C until a constant weight was achieved; about 0.5 g of dried tissues were mixed with 2 mL perchloric acids and 10 mL nitric acids. After digestion at room temperature for 10 min, the levels of Ca, K, and Mg were analyzed using an IPC-OES (optima 8000, Perkin Elmer, Cambridge, MA, USA) according to Falchi et al. [28]. The concentrations of measured mineral elements were expressed by the relative content of dry weight. Three replicates were performed for each sample.

### 4.5. Analysis of Gene Expression

Total RNA was extracted with RNA prep Pure Plant Kit (Polysaccharides & Polyphenolics-rich, Tiangen, China) according to the manufacturer’s instructions. RNA quality was checked by both optical density (OD) value (1.9 to 2.1) at 260/280 nm (NanoDrop 2000C, Thermo Scientific, Waltham, MA, USA) and electrophoresis in a 1.5% agarose gel with sharp, clear 28s and 18s rRNA bands stained by ethidium bromide according to [29]. cDNA was synthesized using the HiScript III RT SuperMix for qPCR (+ gDNA wiper) (Vazyme, Nanjing, China) according to the manufacturer’s instructions, followed by a five-time dilution to be used as the qPCR template. qPCR was performed in a 20 μL reaction system including 1 μL of cDNA, 0.8 μL of each primer, 10 μL of 2 × chamQ SYBR Color qPCR Master Mix (Vazyme, Nanjing, China) and 7.4 μL of water, which was conducted on a Light Cycler^®^ 480 instrument (Roche, Basel, Switzerland). Gene-specific primers were designed (Table S1) and an actin gene was used as the reference gene for the normalization of the transcript level. Two controls of no-RT and no-template were included. Transcript levels were determined using the 2^−ΔΔCt^ method [30].

### 4.6. Localization of Free Ca^2+^

The localization of free Ca^2+^ was analyzed by fluorescence imaging as previously described by Qiu et al. [31] with some modifications. Briefly, thin slices of flesh were collected from the outer flesh tissues of both NI-treated and control fruit using a razor blade. The flesh tissues were initially washed twice on a glass slide with PBS (pH = 7.4) buffer solution, which were loaded with Fluo-4/AM at 37 °C for 1 h and then subsequently washed three times with PBS buffer solution. The Fluo-4 fluorescence maintained on the tissue was visualized under 494 nm excitation wavelength of laser light and 516 nm long-pass emission filter using a laser scanning confocal microscope (TCSSP5Ⅱ, Leica, Wetzlar, Germany). Ten replicates were performed for each sample.

### 4.7. Statistical Analysis

The statistical analysis was performed using GraphPad Prism 8 software (GraphPad Software Inc., San Diego, CA, USA). Significant difference was determined by Duncan’s multiple range test, and significance was tested at *p* < 0.05 or *p* < 0.01 level.

## 5. Conclusions

**Figure 8:**
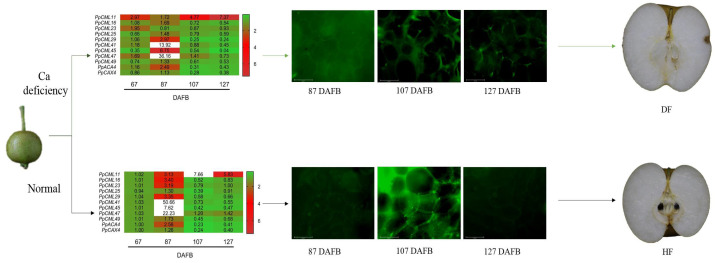
A hypothetical summarization of the relationship between Ca^2+^ deficiency and cork spot occurrence. NI treatment caused the Ca deficiency in the outer flesh and fruit peel at an early stage of the fruit development (67 DAFB). Expression levels of Ca^2+^ transport-related genes then were altered, which further promoted the intracellular imbalance of Ca^2+^ distribution and prompted the cork spot development. DF means cork spot disordered fruit; HF means healthy fruit.

NI treatment promoted the development of cork spot in ‘Akituki’ pear fruit by altering the expression levels of Ca^2+^ transport-related genes; the Ca contents in the outer flesh and peel, especially at early stage (67 DAFB); and finally, the intracellular distribution of Ca^2+^ in certain tissues, as summarized in Figure 8. As a physiological disorder, cork spot is affected by genotypes, environmental factors and management strategies. In areas with high incidences of cork spot, cultivars with lower susceptibility to cork spot should be utilized. Drought stress affects the soil structure, soil pH, organic matter and so on, which all influence the Ca^2+^ uptake of the root; therefore, appropriate irrigation should be fulfilled in the areas with hot summer and less rainfall. Measures such as foliar spraying of a Ca^2+^ solution to prevent and control the occurrence of cork spot should be conducted as early as possible. Enough Ca up-take at an early stage of the fruit development is essential to secure a normal Ca concentration and distribution and reduce the cork spot incidence. In a future study on cork spot disorder, advanced technologies such as multi-omics analysis should be applied to explore the mechanism of cork spot occurrence at the molecular level and help propose more effective control strategies.

## Figures and Tables

**Table 1:** Fruit quality analysis of control and NI-treated ‘Akituki’ fruit. Both the control and NI-treated fruit selected for analysis were healthy fruit without any cork spot.

Treatments	Fresh Weight (g)	Vertical Length (cm)	Horizontal Length (cm)	Total Soluble Solids (%)	Titratable Acidity (%)	Firmness (N)
Control	449.9 ± 28.7 a	10.5 ± 0.4 a	11.2 ± 0.5 a	12.8 ± 0.8 a	0.3 ± 0.02 a	9.8 ± 0.9 a
NI-treated	465.0 ± 94.4 a	10.6 ± 0.6 a	11.6 ± 0.8 a	13.1 ± 0.4 a	0.2 ± 0.03 a	10.7 ± 1.3 a

Data are presented as means ± SE. The same letter in the same column means no significant difference.

## Data Availability

The data presented in this study are available in the article or Supplementary Material.

## Supplementary Materials

The following are available online at https://www.mdpi.com/article/10.3390/plants10050994/s1. Table S1: Genes selected for expression analysis and primers used for RT-qPCR.
