# Supplementary material for: Nitrendipine-Treatment Increases Cork Spot Disorder Incidence in Pear ‘Akituki’ (Pyrus pyrifolia Nakai.) by Altering Calcium Distribution Inside the Fruit
Source: Plants (Basel). 2021 May 17;10(5):994. doi: 10.3390/plants10050994 (PMC8155913; doi:10.3390/plants10050994)
Supplement: Supplementary file 1 [file plants-10-00994-s001.zip › plants-1182547-supplementary.pdf]

plants

Article

Nitrendipine-Treatment Increases Cork Spot Disorder Incidence in Pear 'Akituki' (*Pyrus pyrifolia* Nakai.) by Altering Calcium Distribution Inside the Fruit

Zhenhua Cui †, Nannan Wang †, Dingli Li, Ran Wang and Chunhui Ma *

[1] College of Horticulture, Qingdao Agricultural University, Qingdao 266109; China; zhcui@qau.edu.cn (Z.C.); wangnannan2001@163.com (N.W.); lidingli@qau.edu.cn (D.L.); rwang@qau.edu.cn (R.W.)
* Correspondence: machunhui2000@163.com
† These authors contributed equally to this work.

Table S1. Genes selected for expression analysis and primers used for RT-qPCR.

Gene name/description	Primer name	Primer sequence
PpCML11	PpCML11-F	5′- GGTCCGACATTCAAGGAGAT -3′
/Probable calcium-binding protein	PpCML11-R	5′-AATCTCCCTCCCCCTTAGTT-3′
PpCML16	PpCML16-F	5′-GGGAGCTCTCCGATATGATG-3′
/ Probable calcium-binding protein	PpCML16-R	5′-CGCGAATTCGTTGAAGCTAA-3′
PpCML23	PpCML23-F	5′-AGTTCGCCGAGATCATCAAT-3′
/ Probable calcium-binding protein	PpCML23-R	5′-TGCAGCTCACTTTTGGAGAT-3′
PpCML25	PpCML25-F	5′-TTATCGTCAACCGTCCAGAG-3′
/ Probable calcium-binding protein	PpCML25-R	5′-GATTTTCCCGTCCCCATTTG-3′
PpCML29	PpCML29-F	5′-CTGAGCATCGAGGAGTTCTT-3′
/ Probable calcium-binding protein	PpCML29-R	5′-AGCAATAATCCCCTGGCAAT-3′
PpCML41	PpCML41-F	5′-ACTGCTCGAACCGGAATAAT-3′
/ Probable calcium-binding protein	PpCML41-R	5′-ACATGTACTCACCGATGGAC-3′
PpCML45	PpCML45-F	5′-GCTTTCCTCCAATCCCAAGA-3′
/ Probable calcium-binding protein	PpCML45-R	5′-AAACAGCCCTGTAAGCTCAT-3′
PpCML47	PpCML47-F	5′-AGGCAGCGGAGATTAGTTAC-3′
/ Probable calcium-binding protein	PpCML47-R	5′-GTTCTCATCAAACACGCGAA-3′
PpCML49	PpCML49-F	5′-CTTCATCGACGACAAGGAGA-3′
/ Probable calcium-binding protein	PpCML49-R	5′-CCCCTCCAACTTTGAAGACT-3′
PpACA4	PpACA4-F	5′-CTGTGGCAGAGAAGGAGAC-3′
/H/ACA ribonucleoprotein complex subunit 4-like	PpACA4-R	5′-TCTCTGTGGCATCCTCTAGT-3′
PpCAX4	PpCAX4-F	5′-GGAGAAGAGGAGGAGGAGAA-3′
/CAX-interacting protein 4-like	PpCAX4-R	5′-GAATCAGATGGTGTTGCTGC-3′
PpActin	PpActin-F	5′-CCCAGAAGTGCTCTTCCAAC-3′
/reference gene	PpActin-R	5′-TTGATCTTCATGCTGCTTGG-3′
